# Video-based tools for surgical quality assessment of technical skills in laparoscopic procedures: a systematic review

**DOI:** 10.1007/s00464-023-10076-z

**Published:** 2023-04-26

**Authors:** Alexander A. J. Grüter, Annabel S. Van Lieshout, Stefan E. van Oostendorp, Sofie P. G. Henckens, Johannes C. F. Ket, Suzanne S. Gisbertz, Boudewijn R. Toorenvliet, Pieter J. Tanis, Hendrik J. Bonjer, Jurriaan B. Tuynman

**Affiliations:** 1 Department of Surgery, Amsterdam UMC Location Vrije Universiteit Amsterdam, De Boelelaan 1117, Amsterdam, The Netherlands; 2 Cancer Center Amsterdam, Treatment and Quality of Life, Amsterdam, The Netherlands; 3 Department of Surgery, Rode Kruis Ziekenhuis, Vondellaan 13, Beverwijk, The Netherlands; 4 Department of Surgery, Amsterdam UMC Location University of Amsterdam, Meibergdreef 9, Amsterdam, The Netherlands; 5 Medical Library, Amsterdam UMC, Vrije Universiteit Amsterdam, Amsterdam, The Netherlands; 6 Department of Surgery, Ikazia Hospital, Montessoriweg 1, Rotterdam, The Netherlands; 7 Department of Surgical Oncology and Gastrointestinal Surgery, Erasmus MC, Doctor Molewaterplein 40, Rotterdam, The Netherlands

**Keywords:** Surgical quality assessment, SQA, Laparoscopy, Video-based, Technical skills, Assessment tool

## Abstract

**Background:**

Quality of surgery has substantial impact on both short- and long-term clinical outcomes. This stresses the need for objective surgical quality assessment (SQA) for education, clinical practice and research purposes. The aim of this systematic review was to provide a comprehensive overview of all video-based objective SQA tools in laparoscopic procedures and their validity to objectively assess surgical performance.

**Methods:**

PubMed, Embase.com and Web of Science were systematically searched by two reviewers to identify all studies focusing on video-based SQA tools of technical skills in laparoscopic surgery performed in a clinical setting. Evidence on validity was evaluated using a modified validation scoring system.

**Results:**

Fifty-five studies with a total of 41 video-based SQA tools were identified. These tools were used in 9 different fields of laparoscopic surgery and were divided into 4 categories: the global assessment scale (GAS), the error-based assessment scale (EBAS), the procedure-specific assessment tool (PSAT) and artificial intelligence (AI). The number of studies focusing on these four categories were 21, 6, 31 and 3, respectively. Twelve studies validated the SQA tool with clinical outcomes. In 11 of those studies, a positive association between surgical quality and clinical outcomes was found.

**Conclusion:**

This systematic review included a total of 41 unique video-based SQA tools to assess surgical technical skills in various domains of laparoscopic surgery. This study suggests that validated SQA tools enable objective assessment of surgical performance with relevance for clinical outcomes, which can be used for training, research and quality improvement programs.

**Supplementary Information:**

The online version contains supplementary material available at 10.1007/s00464-023-10076-z.

Optimizing surgical procedures by improving the technique and implementation of innovations have shown to improve clinical outcomes. This indicates that a surgical procedure is evolving over time, and can be performed with varying technique and surgical quality. Awareness of varying surgical quality has major implications for evaluating surgical performance in daily clinical practice as well as determining the impact of surgery on different clinical parameters in a research setting. However, most comparative studies in surgery are hampered by lack of quality assurance which might underestimate the clinical impact of a new surgical innovation, or might influence its relative contribution in multimodality treatment approaches (e.g. added value of perioperative chemotherapy). It has been shown that the quality of surgery has substantial impact on clinical outcomes which is also reflected by suboptimal outcomes in surgical learning curves [1–5].

Currently, surgical competency is not objectively measured in clinical practice using surgical quality assessment (SQA) tools. In surgical education, the competency of a resident to perform a specific operation independently is generally based on subjective rather than objective assessments. Since the evidence of the association between technical skills and patient outcomes is growing, the surgical community as well as health care organizations are seeking solutions to objectively measure a surgeon’s competence and avoid negative impact of variation and learning curves. Objective competence assessment is needed to improve the quality of surgery. This will lead to better performance adjusted surgical education, accommodate the certification of surgeons after successful training and help to obtain robust data in clinical trials investigating new surgical techniques.

Many different tools have been developed for surgical assessments: direct assessment in the operating room by an expert or supervisor, self-assessment after a surgical procedure and postoperative video-based assessment. Especially in laparoscopic surgery, multiple video-based SQA tools have been described, which can be divided in four main categories: (1) global assessment scales (GAS) focusing on overarching qualities such as tissue handling [6, 7], (2) error-based assessment scales (EBAS) in which errors are identified as a surrogate for the overall quality of the performance [8], (3) procedure-specific assessment tools (PSAT) in which key steps and phases of the operation are assessed separately [9], and (4) artificial intelligence (AI) machine learning algorithms which can recognize anatomical structures and movements of instruments to estimate or predict surgical quality [10].

Although many of these video-based SQA tools have been thoroughly investigated, validation of these tools remains complex [11]. Since the increasing need for SQA for education and clinical trial purposes, we aim to provide a clear overview of the available video-based SQA tools, their relation to clinical outcomes and evidence on their validity.

## Methods

### Protocol and registration

This systematic review was conducted in compliance with the guidance from the Preferred Reporting Items for Systematic Reviews and Meta-Analyses (PRISMA) checklist [12]. This study including the review protocol are registered in PROSPERO (ID: 313,008).

### Search strategy

PubMed, Embase.com and Web of Science were systematically searched by two reviewers (AG and AvL) from inception up to September 1st 2022 with the aid of a medical information specialist. The search strategy was created using terminology from studies that met the inclusion criteria, and was primarily focused on laparoscopic surgery, quality assessment tools of technical skills, video-based evaluation and tool validation. Details of the search strategies are provided in Supplementary Tables 1a–c. References of included studies were screened to search for other eligible studies.

### Inclusion and exclusion criteria

Studies were included if video-based quality assessment of laparoscopic surgery in living patients was evaluated. No restrictions regarding type of research methodology was used. All domains of laparoscopic surgery were considered.

Studies were excluded if the focus was on endoscopic (i.e. endoluminal) procedures or robot-assisted procedures and if surgery was performed in the context of a box trainer or virtual reality (VR) setting. Non-human studies, reviews, comment letters and articles written in a language other than English or Spanish were also excluded.

### Selection process and data extraction

Two reviewers (AG and AvL) selected the articles independently after removal of duplicates by screening title and abstract. Subsequently, they independently assessed the remaining potential articles in full text, including their potential relevant references. Discrepancies between the reviewers were discussed and resolved by consensus with a third person (JT). By using a data extraction template, AG and AvL independently extracted pre-defined characteristics of the identified studies, including study design, type of surgical procedure, number of videotaped procedures, number of surgeons, number of patients, name of the tool, number of reviewers, validation approach, results of validation and inter-rater reliability.

### Validation methods and assessment of validity

All methods of validation were identified. Subsequently, the four most common validation methods were selected for analysis, which comprised validation by clinical patient outcomes, validation by experience level of surgeons, validation by expert opinion and validation using another available assessment tool.

In addition, all studies were rated by the same two reviewers (AG and AvL) for evidence of validity using a scoring system provided by Beckman et al. [13], which was later adjusted by Ghaderi et al. [11] and Haug et al. [14]. That scoring system was further modified for the purpose of this systematic review, thereby defining five dimensions of validity: content validity, response process, internal structure, relations to other variables and consequences (see Table 1)*.* All included studies were rated for each dimension with a score from 0 to 3, which could count up to a total score of 15. A score of 1–5 is associated with limited validity, a score of 6–10 with moderate validity and 11–15 with substantial validity. The five domains of our validity evidence scoring list represent the subtypes of the concept ‘validity’ in which one domain is not superior to another. Therefore, these domains weighted equally when calculating the total validity scores. Supplementary Table 2 shows the individual scores per item for all the included articles separately.

**Table 1:** Validity evidence scoring list, adopted from Beckman et al. [13], Ghaderi et al. [11] and Haug et al. [14], and modified for this review

Domain	Definition	Score	Description	Examples
Content validity	The extent to which the tool’s content relates to the construct it intends to measure	0	No data regarding the content validity	
		1	Expert judgment with limited data regarding the tool content	Expert judgment
		2	Listing assessment items for the tool content with some references to a panel of experts, limited description of the developing process References to a previously validated tool	Structured task analysis, hierarchical task analysis Based on previously validated tools
		3	Well-defined developing process, both theoretical basis for the chosen items and systematic review by experts	Delphi method, pilot study
Response process	The analysis of the responses given by the individual assessors and interpretation of the reported results	0	No data regarding the response process	
		1	Limited data reported. Use of an assessment tool without discussing the impact of the differences in response processes	User manuals
		2	Some data regarding different responses of assessors. Some data about systems that reduce variation between respondents	Structured assessor training before the assessment process
		3	Multiple sources of data examining response error through critical examination of response processes and respondents	Validation of initial scores (pilot study), evaluation of response error after structured assessor training
Internal structure	The extent to which individual items describe the underlying constructs, often reported by measures of inter-rater reliability, internal consistency and generalizability	0	No data regarding the internal structure	
		1	Limited data regarding internal structure, references to a single inter-rater reliability measure	Simple measures of inter-rater reliability (ICC, G-theory or Cronbach alpha) *or* inter-item-reliability
		2	A few measures of reliability reported, insufficiently item analysis	Inter-rater reliability coefficient combined with a single measure of inter-item or inter-test reliability
		3	Multiple measures of reliability including interrater reliability and item-analysis (interitem reliability, inter-test reliability, item response theory)	Generalizability theory analysis, item response theory
Relations to other variables	Correlation between assessment scores and other outcomes or scoring systems relevant to the construct being measured	0	No data regarding the other variables	
		1	Correlation of assessment scores with experience or another tool	Tool validated by experience or another tool
		2	Correlation of assessment scores with experience and another tool	Tool validated by experience and another tool
		3	Correlation between assessment scores and clinical outcomes	Tool validated by clinical outcomes
Consequences	The impact of the assessment and future use	0	No data regarding the consequences	
		1	Limited data, merely a discussion about future use	Describing feasibility and potential future use (data on assessment time, post assessment survey)
		2	Some descriptions of consequences of assessment for learners, often supported by incomplete data	Describing educational impact (formative/summative feedback, learning curve of trainees)
		3	Clear description of consequences of assessments and the impact on interpretation of scores and intended future use, supported by data	Criterion-referenced score (pass/fail-scores), cut-off scores for licensing purposes, predictive models

## Results

### Literature search

**Fig. 1:**
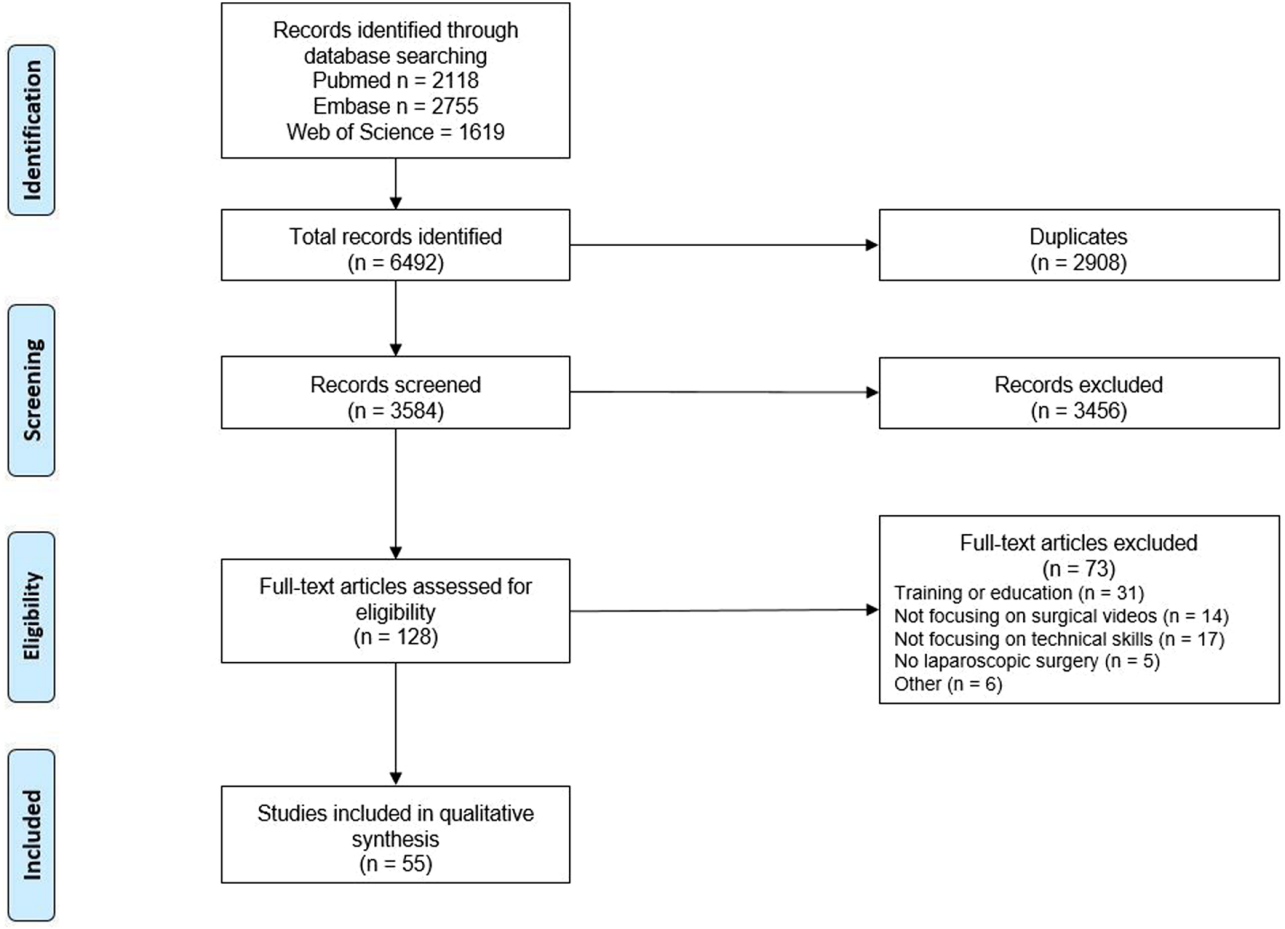
PRISMA flowchart of the literature search

The literature search yielded 6492 records that resulted in 3584 unique articles after removal of duplicates. After title and abstract screening, 128 full text articles were assessed. A total of 73 articles were excluded for reasons as outlined in Fig. 1, which resulted in 55 studies [1–3, 8, 9, 15–64]. An overview of the included studies is provided in Table 2.

**Table 2:** Overview of the included studies

Kind of assessment	#	Author	Year	Surgical procedure	Number of videos	Whole operation (WH) or parts (P)	Number of surgeons	Name of tool/AI	Number of reviewers	Validation of tool to clinical outcomes (CO), experience (EXP), another tool (AT) or experts opinion (EO)
Global assessment scale (GAS)	1	Varban [61]	2021	Laparoscopic sleeve gastrectomy and gastric bypass	25	WH	25	BOSATS	25	CO
	2	Varban [62]	2021	Laparoscopic sleeve gastrectomy	33	WH	25	BOSATS	25	CO
	3	Chhabra [21]	2021	Laparoscopic sleeve gastrectomy	46	P	30	BOSATS	25	CO
	4	Fecso [26]	2019	Laparoscopic gastrectomy	61	WH	3	OSATS & GERT	1	CO
	5	Goderstad [29]	2016	Laparoscopic supracervical hysterectomy	37	WH	23	GOALS & CAT-LSH	2	EXP, AT & EO
	6	Scally [55]	2016	Laparoscopic gastric bypass	20	WH	20	OSATS	NA	CO
	7	Kramp [42]	2015	Laparoscopic cholecystectomy	3	WH	3	ISPA, OSATS & GOALS	19	EXP & EO
	8	Koehler [40]	2015	Diagnostic portion of routine shoulder and knee arthroscopic procedures	70	WH	12	ASSET	2	None
	9	Kramp [41]	2015	Laparoscopic cholecystectomy	60	WH	10	GOALS	2	AT
	10	Kasparian [36]	2014	Laparoscopic cholecystectomy and Lichstenstein's inguinal hernia repair	67	WH	62	OSATS	2	EXP
	11	Matsuda [47]	2014	Laparoscopic adrenalectomy or laparoscopic nephrectomy	1220	WH	787	ESSQ	42	None
	12	Birkmeyer [3]	2013	Laparoscopic gastric bypass	20	P	20	BOSATS	33	CO
	13	Koehler [39]	2013	Diagnostic knee arthroscopy	60	WH	30	ASSET	2	EXP
	14	Oestergaard [50]	2012	Right side laparoscopic salpingectomy	3	WH	3	OSA-LS	20	EXP
	15	Herati [33]	2012	Laparoscopic radical or partial nephrectomy	32	P	11	GRS, ORS & CRS	4	EXP
	16	Larsen [44]	2008	Right side laparoscopic salpingectomies	21	WH	21	OSA-LS	2	EXP
	17	Aggarwal [15]	2008	Laparoscopic cholecystectomy	47	WH	19	OSATS	2	AT & EXP
	18	Aggarwal [16]	2007	Laparoscopic cholecystectomy	54	WH	19	OSATS	2	EXP
	19	Chang [19]	2007	Laparoscopic cholecystectomy	2	WH	2	GOALS	10	EXP
	20	Vassiliou [63]	2007	Laparoscopic cholecystectomy	10	WH	10	GOALS	4	EXP
	21	Shime [56]	2003	Gynaecologic laparoscopic operations	20	WH	20	LSI	4	EO
Error-based assessment scale (EBAS)	4	Fecso [26]	2019	Laparoscopic gastrectomy	61	WH	3	OSATS & GERT	1	CO
	22	Foster [27]	2016	Laparoscopic rectal cancer surgery (TME and ELAPE)	20	WH	NA	OCHRA	1	CO
	23	Husslein [34]	2015	Laparoscopic hysterectomy	20	WH	14	GERT	2	EXP & AT
	24	Bonrath [8]	2013	Laparoscopic Roux-en-Y gastric bypass	25	WH	NA	GERT	2	AT
	25	Miskovic [49]	2012	Right and left colectomies	32	WH	21	OCHRA	2	AT
	26	Tang [58]	2004	Laparoscopic pyloromyotomy	50	WH	5	OCHRA	1	None
Procedure-specific assessment tool (PSAT)	27	Haug [32]	2022	Laparoscopic right and left colectomy (complete mesocolic excision)	NA	WH	NA	CMECAT	NA	EO
	28	Sirimanna [57]	2022	Laparoscopic appendectomy	18	WH	18	LARS	2	EXP, AT
	29	Chevallay [20]	2022	Laparoscopic cholecystectomy	42	WH	15	LCAT	3	EO
	30	Kurashima [43]	2022	Laparoscopic distal gastrectomy	54	WH	40	JORS-LDG	3	CO
	31	Harris [31]	2022	Esophagectomy	31	WH	NA	Two-stage esophagectomy video assessment tool	3	None
	32	Kobayashi [38]	2022	Laparoscopic hysterectomy	46	WH	NA	Modified OSATS	29	CO
	33	Dixon [24]	2021	Laparoscopic gastrectomy	10	P	NA	KLASS guideline	4	None
	34	Crochet [22]	2021	Laparoscopic hysterectomy	217	P	NA	H-OSATS	2	EXP
	35	Han [30]	2021	Open and laparoscopic distal gastrectomies	159	WH	27	Video assessment form	5	EXP & EO
	36	Stulberg [1]	2020	Laparoscopic right hemicolectomy	17	WH	17	OSATS & COSATS	17	CO
	37	Varban [60]	2020	Laparoscopic sleeve gastrectomy	30	WH	30	OSGS	52	CO
	38	Curtis [2]	2020	Laparoscopic TME	176	WH	34	Performance tool	1	CO
	39	Tsai [59]	2019	TaTME	NA	WH	14	CAT-tool	14	EO
	40	Ki Bum Park [51]	2019	Laparoscopic appendectomy	100	WH	NA	Appendectomy scoring system & GOALS	NA	AT
	41	Savran [54]	2019	Laparoscopic hysterectomy	16	WH	16	Rating scale	NA	EXP
	42	Jensen [35]	2018	VATS lobectomy	NA	WH	28	VATSAT	NA	None
	43	Petersen [52]	2018	VATS lobectomy	60	WH	18	VATSAT	2	EO
	44	Champagne [18]	2017	Laparoscopic right hemicolectomy	24	WH	NA	ASCRS Tool	20	EO
	45	Deal [23]	2017	Laparoscopic cholecystectomy	160	P	NA	CVS assessment tool	5	AT
	5	Goderstad [29]	2016	Laparoscopic supracervical hysterectomy	37	WH	23	CAT-LSH & GOALS	2	EXP, AT & EO
	7	Kramp [42]	2016	Laparoscopic cholecystectomy	3	WH	3	IPSA, OSATS & GOALS	19	EXP & EO
	46	Poudel [53]	2016	TAPP (transabdominal peritoneal procedure)	30	WH	NA	TAPP checklist & GOALS-GH	3	AT, EO
	47	Mackenzie [46]	2015	Laparoscopic right and left hemicolectomy	171	WH	85	CAT-tool	2	CO & EO
	48	Miskovic [48]	2013	Colorectal surgery	54	WH	31	CAT tool	2	EXP
	49	Zevin [64]	2013	Laparoscopic gastric bypass	52	WH	NA	BOSATS	2	EXP
	14	Oestergaard [50]	2012	Right side laparoscopic salpingectomy	3	WH	3	OSA-LS	20	EXP
	50	Palter [9]	2012	Laparoscopic right and sigmoid colectomy	37	WH	23	Procedure-specific technical skills evaluation tool	2	EXP
	15	Herati [33]	2012	Laparoscopic radical or partial nephrectomy	32	P	11	GRS, ORS & CRS	4	EXP
	16	Larsen [44]	2008	Right side laparoscopic salpingectomies	21	WH	21	OSA-LS	2	EXP
	51	Eubanks [25]	1999	Laparoscopic cholecystectomy	30	WH	30	The scoring system	3	EXP
	52	Beckmann [17]	1995	Laparoscopic tubal banding	23	WH	NA	Surgical skill checklist	7	EO
Artificial Intelligence (AI)	53	Kitaguchi [37]	2021	Laparoscopic sigmoid resection	650	WH	NA	3-Dimensional Convolutional Neural Network	NA	AT
	54	Lavanchy [45]	2021	Laparoscopic cholecystectomy	242	P	NA	Convolutional Neural Network	NA	EO
	55	Ganni [28]	2020	Laparoscopic cholecystectomy	12	WH	12	Kinovea 0.8.15 software	NA	AT & EXP

*ASCRS*: American Society of Colon and Rectal Surgeons; *ASSET*: Arthroscopic Surgery Skill Evaluation Tool; *BOSATS*: Bariatric Objective Assessment of Technical Skill; *CAT*: Competency Assessment Tool; *CAT-LSH:* Competency Assessment Tool Laparoscopic Supracervical Hysterectomy; *CMECAT*: Complete Mesocolic Excision Competency Assessment Tool; *COSATS:* Colorectal Objective Structured Assessment of Technical Skill*; CRS*: Cognitive Rating Scale; *CVS*: Critical View of Safety; *ESSQ*: Endoscopic Surgical Skill Qualification; *GERT:* Generic Error Rating Tool*; GOALS:* Global Operative Assessment of Laparoscopic Skills*; GOALS-GH*: Global Operative Assessment of Laparoscopic Skills-Groin Hernia; *GRS*: Global Rating Scale; *H-OSATS*: Hysterectomy-Objective Structured Assessment Technical Skills; *ISPA:* independence-scaled procedural assessment; *JORS-LDG*: Japanese Operative-Rating Scale for Laparoscopic Distal Gastrectomy; *LARS*: Laparoscopic Appendectomy Rating Scale; *LCAT*: Laparoscopic Competency Assessment Tool; *LSI*: Laparoscopic Skills Index; *OCHRA*: Observational Clinical Human Reliability Assessment; *ORS*: Operation-Specific Rating Scale; *OSA-LS*: Objective Structured Assessment of Laparoscopic Salpingectomy; *OSATS:* Objective Structured Assessment Technical Skills*; OSGS*: Optimal Sleeve Gastrectomy Score; *TAPP*: Transabdominal Preperitoneal Procedure; *VAS*: Visual Analogue Scale; *VATSAT*: Video-Assisted Thoracoscopic Surgery Assessment Tool

### Characteristics of the assessment tools

The literature search identified 55 articles, which presented 41 different video-based tools for technical skills assessment in 9 different fields of surgery including bariatric, gynecologic, general, upper gastrointestinal, orthopedic, urologic, colorectal, pediatric and pulmonary surgery (see Table 2). Described SQA tools could be divided into four main categories: “Global assessment scale (GAS)” was investigated in 21 studies [1, 15, 16, 19, 21, 26, 29, 33, 36, 39–42, 44, 47, 50, 55, 56, 61–63], “Error-based assessment scale (EBAS)” was investigated in 6 studies [8, 26, 27, 34, 49, 58], “Procedure-specific assessment tool (PSAT)” was investigated in 31 studies [2, 3, 9, 17, 18, 20, 22–25, 29–33, 35, 38, 42–44, 46, 48, 50–54, 57, 59, 60, 64] and 3 studies examined the use of “Artificial Intelligence (AI)” [28, 37, 45].

In total, 12 articles focused on the correlation between the assessment score and clinical outcomes of which 8 were performed in bariatric surgery and 4 in colorectal surgery (Table 3). A total of 26 tools were validated based on the experience level of surgeons. In most studies, assessment scores of experienced surgeons were compared with the scores of surgeons with an intermediate or beginners level (often surgical residents), based on either their years of practice or number of performed procedures. A total of 12 studies validated their assessment tool by another available assessment tool, with the vast majority using the Objective Structured Assessment of Technical Skills (OSATS) or Global Operative Assessment of Laparoscopic Skills (GOALS) as a comparative scale. Expert opinion was used in 15 studies to validate their assessment tool.

**Table 3:** Overview of studies with assessment tools validated by clinical outcomes

#	Author	Journal & Year	Surgical procedure	Name of tool (type of tool)	Observed clinical outcomes	Amount of observed patients for clinical outcomes	Statistically significant correlation with clinical outcomes	Groups and cut-off values based on assessment scores
1	Kurashima [43]	Surg Endosc. 2022	Laparoscopic distal gastrectomy	JORS-LDG (PSAT)	Operation time, number of harvested lymph node, haemorrhage, intraoperative complications, postoperative complications, postoperative stay	54	- Median operation time (229 vs. 266 vs. 311 min, *P* < 0.001) - Intraoperative complication rate (0% vs. 11.8% vs. 27.8%, *P* = 0.01) - Postoperative complication rate (0% vs. 0% vs. 22.2%, *P* = 0.002)	High (JORS-LDG score of 42–44), intermediate (JORS-LDG score of 39–41) or low performance (JORS-LDG score of ≤ 38)
2	Varban [61]	Ann Surg 2021	Laparoscopic sleeve gastrectomy and gastric bypass	BOSATS (GAS)	Any complication, surgical complication, infection, leak, hemorrhage, stricture, reoperation, mortality (30 days after operation)	37,074	Leak rates (0.27% vs. 0.65%, *P* = 0.0181)	Highest quartile vs. lowest quartile based on BOSATS score of one video
3	Varban [62]	Ann Surg 2021	Laparoscopic sleeve gastrectomy	BOSATS (GAS)	Leak, obstruction, infection, hemorrhage, venous thromboembolism, cardiac complications, pulmonary complications, death, reoperation, readmission, ED visit (all 30 days after operation), EBWL% (1 year after operation)	3607	Postoperative obstruction (0.13% vs. 0.3%, *P* = 0.017), hemorrhage (0.85% vs. 1.27%, *P* = 0.005), reoperation (0.24% vs. 0.92%, *P* < 0.0001), %EBWL (58.5% vs. 56.1%, *P* = 0.03)	Highest quartile vs. lowest quartile based on BOSATS score of one video
4	Chhabra [21]	JAMA Surg 2021	Laparoscopic sleeve gastrectomy	BOSATS (GAS)	Hemorrhage, leak, weight loss, patient-reported reflux severity	6915	- Hemorrhage was statistically significantly correlated with 4 of the 5 steps (1.0% vs. 2.1%, *P* = 0.01; 0.94% vs. 1.5%, *P* = 0.006; 1.2% vs. 2.8%, P = 0.03; *P* = 0.049) - Leak rates were statistically significantly correlated with 3 of the 5 septs (0.16% vs. 0.05%, *P* < 0.001; 0.2% vs. 0.1%, *P* = 0.003; 0.1% vs. 0.02%, *P* = 0.01; 0.18% vs. 0.07%, *P* < 0.001) - Weight loss was statistically significantly correlated with 3 of the 5 steps (28.7% vs. 27.1%, *P* = 0.02; 28.9% vs. 27.7%, *P* = 0.03; 28.0% vs. 24.9%, *P* = 0.02) - Patient-reported reflux severity was statistically significantly correlated with 4 of the 5 steps (− 1.3 vs. − 0.16, *P* < 0.001; − 1.5 vs. − 0.8, *P* = 0.006; − 1.0 vs. 0.8, *P* = 0.001; − 1.3 vs. − 2.0, *P* = 0.002)	Highest quartile vs. lowest quartile based on BOSATS score based on one or two videos
5	Stulberg [1]	JAMA Surg 2020	Laparoscopic right hemicolectomy	OSATS + COSATS combined (PSAT)	Any complication, mortality, unplanned readmission, unplanned reoperation, SSI, death or serious morbidity	3063	Any complication (15.5% vs. 20.6%, *P* = 0.03), unplanned reoperation (4.7% vs. 7.2%, *P* = 0.02), death or serious morbidity (15.9% vs. 21.4%, *P* = 0.02)	Highest quartile vs. lowest quartile based on combination of OSATS and COSATS of one laparoscopic right hemicolectomy video
6	Curtis [2]	JAMA Surg 2020	Laparoscopic TME	Performance tool (PSAT)	Circumferential margin ≥ 1 mm, distal margin ≥ 1 mm, lymph node yield, overall survival, recurrence data, 30-day morbidity, operation duration, blood loss, unplanned reoperation, anastomotic leak, length of stay, readmission	176	30-day morbidity (23.3% vs 55.3% vs. 50%, *P* = 0.008), operation duration (median 178 min vs. median 255 min. vs. median 290 min, *P* < 0.001), blood loss (median 40 mL vs. median 100 mL vs. median 100 mL, *P* < 0.001)	Upper quartile vs. interquartile vs. lower quartile based on performance tool of every single video/patient
7	Varban [60]	J Am Coll Surg 2020	Laparoscopic sleeve gastrectomy	OSGS (PSAT)	Surgical complication, leak, hemorrhage, reoperation, stricture, excess body weight loss, total body weight loss	7201	Surgical complications (1.54% vs. 2.75%, OR 0.56, 95% CI 0.35–0.88, *P* = 0.013), hemorrhage (0.61% vs. 1.48%, OR 0.49, 95% CI 0.28–0.86, *P* = 0.013), reoperation (0.37% vs. 0.91%, OR 0.4, 95% CI 0.20–0.81, *P* = 0.010)	Upper quartile vs. interquartile vs. lower quartile based on one video
8	Fecso [26]	Ann Surg 2019	Laparoscopic gastrectomy	OSATS & GERT (GAS & EBAS)	Major postoperative complications (death, anastomotic leak, intra-abdominal abscess, internal hernia, intestinal obstruction, single organ dysfunction (respiratory), intra-abdominal bleeding)	61	Major postoperative complications, Clavien-Dindo ≥ III, only statistically significant with OSATS score (OR 6.49, 95% CI 1.60–26.34, *P* = 0.009)	- High-performance group (OSATS score > 29/35) vs. low-performance group (OSATS score ≤ 29/35) based on every single video/patient - Amount of errors, events and rectifications
9	Foster [27]	Tech Coloproctol 2016	Laparoscopic rectal cancer surgery (TME and ELAPE)	OCHRA (EBAS)	30-day postoperative morbidity, operation time, blood loss	20	Total blood loss (r_s_ = 0.61, *P* = 0.004)	Total error frequency of every single video
10	Scally [55]	JAMA Surg 2016	Laparoscopic gastric bypass	OSATS (GAS)	EBWL%, resolution of medical comorbidities (hypertension, sleep apnea, diabetes and hyperlipidemia), functional status, patient satisfaction	3631	None	Highest quartile vs. lowest quartile based on OSATS score of one video
11	Mackenzie [46]	Br J Surg 2015	Laparoscopic right and left hemicolectomy	CAT-tool (PSAT)	Complications, surgical complications, medical complications, lymph node count	171	Postoperative morbidity (8.7 vs. 25%, *P* = 0.005), surgical complications (6.3 vs. 18%, *P* = 0.02), lymph node yield (median 18 vs. median 13, *P* = 0.004)	Pass group (mean score ≥ 2.7) vs fail group (mean score < 2.7) based on two videos
12	Birkmeyer [3]	NEJM 2013	Laparoscopic gastric bypass	BOSATS (GAS)	Leak or perforation, obstruction, infection, hemorrhage, venous thromboembolism, cardiac complication, renal failure, pulmonary complication, death, operation time, reoperation, readmission, return visits to ED	10,343	Complication rates (5.2% vs. 14.5%, *P* < 0.001), mortality (0.05% vs. 0.26%, *P* = 0.01), operation time (98 min vs. 137 min, *P* < 0.001), reoperation (1.6% vs. 3.4%, *P* = 0.01), readmission (2.7% vs. *P* < 0.001)	Highest quartile vs. lowest quartile based on BOSATS score of one video

*BOSATS*: Bariatric Objective Assessment of Technical Skill; *CAT*: Competency Assessment Tool; *COSATS:* Colorectal Objective Structured Assessment of Technical Skill*; CRS*: Cognitive Rating Scale; *GERT:* Generic Error Rating Tool*; GRS*: Global Rating Scale; *JORS-LDG*: Japanese Operative-Rating Scale for Laparoscopic Distal Gastrectomy; *OCHRA*: Observational Clinical Human Reliability Assessment; *OSATS:* Objective Structured Assessment Technical Skills*; OSGS*: Optimal Sleeve Gastrectomy Score; *GAS*: Global assessment scale; *EBAS*: Error-based assessment scale; *PSAT:* Procedure-specific assessment tool; *AI:* Artificial Intelligence; *TME*: total mesorectal excision; *ELAPE*: extralevator abdominoperineal excision

#### Global assessment scale (GAS)

In total, 21 studies investigated an assessment tool that could be categorized as GAS, of which 12 studies used the Objective Structured Assessment of Technical Skills (OSATS) or modified versions of this tool, for example the Bariatric Objective Structured Assessment of Technical Skills (BOSATS). Six studies validated their GAS with clinical patient outcomes, the majority of which were performed in bariatric surgery (see Table 2). Two articles examined whether the quality of surgery resulting from the OSATS correlated with clinical outcomes. The study of Fecso et al. showed that a lower performance score (OSATS ≤ 29/35) was an independent predictor for major-short term outcomes in laparoscopic gastrectomy (OR 6.49, 95% 1.60–26.34, *P* = 0.009) [26]. In contrast, the results of Scally et al. revealed no difference in clinical outcomes between the 75th percentile (25% highest rated surgeons) and the 25th percentile (25% lowest rated surgeons) based on the OSATS score [55]. The other four papers investigated whether BOSATS was correlated with patient outcomes showed conflicting results [1, 21, 61, 62]. In one of these studies, the anastomotic leakage rate was significantly correlated with the technical execution of the operation [61]. In the other two papers, a non-significant association was seen [1, 62]. In contrast, the study of Chhabra et al. showed that higher assessment scores of certain parts of laparoscopic sleeve gastrectomy were associated with increased leakage rates [21]. Three studies evaluated reoperation rates, of which two studies showed a significant correlation of the assessment score with the reintervention rate [1, 61, 62]. In two of the four studies focusing on surgical haemorrhage, a significant correlation was found [21, 62] while in the other two a trend was seen [1, 61]. In Table 3 a detailed overview of all studies with assessment tools validated by clinical outcomes is provided.

#### Error-based assessment scale (EBAS)

A minority of the tools were classified as EBAS. The Objective Clinical Human Reliability Analysis (OCHRA) and the Generic Error Rating Tool (GERT) were mostly used in the literature so far. Both OCHRA and GERT were used in three studies. However, OCHRA was limited to the field of gastrointestinal surgery, while GERT was investigated in bariatric and gynecologic procedures (see Table 2). Two studies looked at the correlation between EBAS and clinical outcomes. In terms of number of errors (*P* = 0.331), events (*P* = 0.758), and rectification (*P* = 0.433), Fecso et al. found no statistically significant difference between the group of patients without complications versus the two groups of patients with either Clavien-Dindo grade I/II or Clavien-Dindo grade III complications. Despite not being significant, it did show a trend with more number of errors, events and rectification in the second group [26]. In addition, Foster et al. did find a statistically significant correlation between total error frequency per case and total blood loss (*rs* = 0.61, *P* = 0.004), measured by OCHRA, [27], see Table 3.

#### Procedure-specific assessment tool (PSAT)

A total of 31 studies assessed surgical procedures with a procedure-specific assessment tool (PSAT). This type of tool has the most variety of tools since these are build based on step-by-step approach dependent on the type of surgical procedure. The most frequently investigated tool is the competency assessment tool (CAT), which was evaluated in three colorectal studies and one gynecological study. In total, five of the PSATs were validated by clinical outcomes (Table 3). In one of those studies, the quality of the surgeon was assessed with both OSATS and a procedure-specific Colorectal Objective Structured Assessment of Technical Skill (COSATS) based on one laparoscopic right hemicolectomy. They compared postoperative complications between the highest quartile and lowest quartile of surgeons and showed that patients operated by surgeons among the highest quartile had fewer complications (15.5% vs. 20.6%, *P* = 0.03), fewer unplanned reoperations (4.7% vs. 7.2%, *P* = 0.02) and lower rates of serious morbidity or death (15.9% vs. 21.4%, *P* = 0.02) compared to patients operated by surgeons belonging to the lowest quartile [3]. In addition, Varban et al. showed that a low PSAT score in a laparoscopic sleeve gastrectomy increased the risk of surgical complications, hemorrhage and reoperation [60]. The study of Karushima et al. focusing on laparoscopic distal gastrectomy also showed a correlation between the PSAT score (high vs. intermediate vs. low) and operative time (229 vs. 266 vs. 311 min, *P* < 0.001), intraoperative complications (0% vs. 11.8% vs. 27.8%, *P* = 0.01) and postoperative complications (0% vs. 0% vs. 22.2%, *P* = 0.002) [43]. Not only in bariatric surgery, but also in colorectal surgery, the association between quality of surgery and clinical outcomes was investigated. Curtis et al. showed a statistically significant difference in 30-day morbidity after laparoscopic total mesorectal excision (TME) between the upper quartile, interquartile and lower quartile (23.3% vs 55.3% vs. 50%, *P* = 0.008), based on a procedure-specific performance tool. Performance was also correlated with operative time (median 178 min vs. 255 min. vs. 290 min, *P* < 0.001) and blood loss (median 40 mL vs. 100 mL vs. 100 mL, *P* < 0.001) [2]. Furthermore, Mackenzie et al. showed that surgeons performing a right or left hemicolectomy with a high assessment score had more favorable patient outcomes: lower postoperative morbidity and surgical complications rates and higher lymph node yield [46], see Table 3.

#### Artificial intelligence (AI)

Three of the included studies used AI to calculate parameters which estimate and predict surgical quality. In one of the studies, videos of laparoscopic cholecystectomy were analyzed by Kinovea 0.8.15 software. Three parameters were calculated: “path length”, “average distance”, which the instrument tip moved per time frame, and “number of extreme movements”, defined as more than 1.0 cm movement per frame. A formula using these parameters calculated a score between 0 and 1, the higher the score the better the execution. Those videos were also scored by a CAT tool and a statistically correlation between both was observed (*R*^2^ = 0.844) [28]. In the other two studies, a convolutional neural network (CNN) was built based on multiple video fragments, which showed to be able to differentiate between different levels or score goups of surgical skills. In the study of Kitaguchi et al., the CNN was able to automatically classify video clips into three different score groups with 75% accuracy, while in the remaining study from Lavancy et al., the CNN could distinguish good from poor quality with an accuracy of 87 ± 0.2% [37, 45].

### Evaluation of validity evidence

The assessment tools and AI in all articles were scored based on the content validity, response process, internal structure, relations to other variables and consequences, as shown in Table 1. The evidence of validity scores for those tools in all articles are presented in Tables 4 and 5. In total, 9 studies received a substantial evidence score (score between 11 and 15), 38 studies were scored as moderate evidence (score between 6 and 10) and the remaining 8 studies were given a limited evidence score (score between 0 and 5). Table 4 shows an overview of all studies and tools arranged by strength of validity based on the validity evidence scoring list from Table 1.

**Table 4:** Articles/tools arranged by strength of validity based on the validity evidence scoring list from Table 1 (substantial, moderate and limited evidence)

Kind of assessment	Article	Tool name	Type of tool	Total
Substantial evidence (score 11–5)	Kramp [42]	ISPA, OSATS & GOALS	GAS + PSAT	12
	Shime [56]	LSI	GAS	11
	Kurashima [43]	JORS-LDG	PSAT	11
	Curtis [2]	Performance tool	PSAT	12
	Stulberg [1]	OSATS & COSATS	PSAT	12
	Petersen [52]	VATSAT	PSAT	11
	Champagne [18]	ASCRS Tool	PSAT	12
	Miskovic [48]	CAT tool	PSAT	12
	Zevin [64]	BOSATS	PSAT	12
Moderate evidence (score 6–10)	Varban [61]	BOSATS	GAS	6
	Varban [62]	BOSATS	GAS	7
	Chhabra [21]	BOSATS	GAS	7
	Fecso [26]	OSATS & GERT	GAS + EBAS	9
	Goderstad [29]	GOALS & CAT-LSH	GAS + PSAT	6
	Scally [55]	OSATS	GAS	8
	Koehler [40]	ASSET	GAS	8
	Kramp [41]	GOALS	GAS	8
	Kasparian [36]	OSATS	GAS	6
	Birkmeyer [3]	BOSATS	GAS	9
	Koehler [39]	ASSET	GAS	10
	Larsen [44]	OSA-LS	GAS + PSAT	8
	Aggarwal [15]	OSATS	GAS	9
	Aggarwal [16]	OSATS	GAS	9
	Vassiliou [63]	GOALS	GAS	9
	Foster [27]	OCHRA	EBAS	7
	Husslein [34]	GERT	EBAS	9
	Bonrath [8]	GERT	EBAS	9
	Miskovic [49]	OCHRA	EBAS	9
	Tang [58]	OCHRA	EBAS	7
	Haug [32]	CMECAT	PSAT	8
	Sirimanna [57]	LARS	PSAT	10
	Chevallay [20]	LCAT	PSAT	7
	Harris [31]	Two-stage esophagectomy video assessment tool	PSAT	7
	Kobayashi [38]	Modified OSATS	PSAT	6
	Crochet [22]	H-OSATS	PSAT	8
	Han [30]	Video assessment form	PSAT	9
	Varban[60]	OSGS	PSAT	6
	Tsai [59]	CAT-tool	PSAT	6
	Savran [54]	Rating scale	PSAT	10
	Deal [23]	CVS assessment tool	PSAT	8
	Poudel [53]	TAPP checklist & GOALS-GH	PSAT	10
	Mackenzie [46]	CAT tool	PSAT	8
	Palter [9]	Procedure-specific technical skills evaluation tool	PSAT	9
	Eubanks [25]	The scoring system	PSAT	9
	Kitaguchi [37]	3-Dimensional Convolutional Neural Network	AI	8
	Lavanchy [45]	Convolutional Neural Network	AI	7
	Ganni [28]	Kinovea 0.8.15 software	AI	8
Limited evidence (score 0–5)	Matsuda [47]	ESSQ	GAS	5
	Oestergaard [50]	OSA-LS	GAS + PSAT	5
	Herati [33]	GRS, ORS & CRS	GAS + PSAT	5
	Chang [19]	GOALS	GAS	4
	Ki Bum Park [51]	Appendectomy scoring system & GOALS	PSAT	5
	Dixon [24]	KLASS guideline	PSAT	3
	Jensen [35]	VATSAT	PSAT	3
	Beckmann [17]	Surgical skill checklist	PSAT	5

**Table 5:** Articles/tools with substantial evidence based on the validity evidence scoring list from Table 1

Articles with substantial validity evidence	Tool name	Type of tool	Content: clear content made by experts (max of 3 points)	Response process: training and analyses of the individual assessors (max of 3 points)	Internal structure: measurements of interrater, interitem or intertest variability (max of 3 points)	Relations to other variables: comparison with clinical outcomes, another tool, experience etc (max of 3 points)
Kramp [42]	ISPA, OSATS & GOALS	GAS + PSAT	2	2	3	2
Shime [56]	LSI	GAS	3	2	3	1
Kurashima [43]	JORS-LDG	PSAT	3	2	1	3
Curtis [2]	Performance tool	PSAT	3	1	2	3
Stulberg [1]	OSATS & COSATS	PSAT	2	3	2	3
Petersen [52]	VATSAT	PSAT	3	2	2	1
Champagne [18]	ASCRS Tool	PSAT	3	2	2	2
Miskovic [48]	CAT tool	PSAT	3	1	3	2
Zevin [64]	BOSATS	PSAT	3	2	3	2
Number of these studies with the maximum score (3/3) per item			7 (77.8%)	1 (11.1%)	4 (44.4%)	3 (33.3%)

In Table 5, all nine studies with substantial validity evidence (score between 11 and 15) and their points per validity item are shown. In total, 7 of the 9 studies (77.8%) received the maximum score of 3 points for clear and accurate content of the tool, by creating the SQA tool using the Delphi method. For the item response process, which reflects the use of training or systems to reduce variation between assessors, only 1 study (11.1%) received the maximum score of 3 points. For the item internal structure representing variability, consistency and generalizability, 4 of the 9 studies (44.4%) received all 3 points. Finally, 3 of the 9 studies (33.3%) scored the maximum of 3 points for the item relation to other variables.

## Discussion

This systematic review shows a comprehensive overview of all video-based SQA tools for technical skills in laparoscopic surgery. In total, 41 tools were identified, which can be divided in four categories: global assessment scale (GAS), error-based assessment scale (EBAS), procedure-specific assessment tool (PSAT), and artificial intelligence (AI). Both PSAT and GAS show the most relevant associations with clinical outcomes. GAS seems more appropriate for general surgical skills during the first training years, while PSAT might be more suitable for evaluating whether someone is able to perform every step of a specific operation accurately. A “good” surgeon based on a GAS does not necessarily mean that he or she is competent to perform a specialized surgical procedure independently. However, before implementing tools in education, clinical practice or research, validation of potential SQA tools is key.

Recently, Haug et al. [14] provided an adequate summary of assessment tools in laparoscopic colorectal surgery, however a clear overview of the available video-based SQA tools in all different fields of laparoscopic surgery including critical evaluation of their validity evidence has not yet been published. Although validation of these tools with experience of surgeons, other tools or expert opinion is interesting, the association between the assessment score and clinical patient outcomes is particularly relevant. Various surgical specialists such as general surgeons, urologists and gynecologists have investigated the value of SQA tools. However, studies that validated SQA with clinical patient outcomes are limited to bariatric and colorectal surgery. In bariatric surgery, a statistically significant positive correlation has been observed between two types of tools (GAS and PSAT) and intra- and postoperative outcomes including decreased anastomotic leakage rates [61], hemorrhage [21, 60, 62], rate of reoperations [60, 62], overall complications [1, 26, 60] and increased percentage of weight loss [21, 62]. The one study investigating EBAS, however, did not show an evident association between its score and clinical patient outcomes [26]. In colorectal surgery, only PSAT and EBAS have been investigated using patient outcomes. Higher PSAT scores seem to be associated with improved patient outcomes including decreased operative time, postoperative morbidity, reoperation, readmission and death [2, 3, 46], while EBAS only showed reduced blood loss [27].

Many studies showed a correlation between high SQA scores and improved clinical outcomes. However, they were heterogeneous and showed moderate validity evidence based on low content quality, no clear training of assessors and high inter-observer variability. The three studies of Kurashima, Curtis and Stulberg, using the JORS-LDG tool (PSAT), the combined tool of OSATS + COSATS (GAS + PSAT) and the Performance Tool (PSAT), respectively, showed both decreased short-term morbidity in case of higher assessment scores and received the best validity scores [2, 3, 65]. These tools for bariatric and colorectal surgery therefore seem the most promising SQA tools at the moment. When looking at the 9 studies with the highest validity (Table 5), it is clear that on some validity items there is room for improvement. Although a high percentage of 77.8% of those articles show high quality of tool content, in 8 of those 9 articles (89.9%) there is no clear response process in which assessors are trained in using this tool, which increases the chance of unwanted variation. In addition, only in 44.4% of those articles optimal internal structure measurements such as inter-rater, inter-item and inter-test variability analyses were performed, and only 33% compared their tool with clinical outcomes. Ideally, an SQA tool achieves maximum scores on all items before implementation: content made by a Delphi consensus with experts (widely used method to achieve consensus on a complex problem) [75], optimal training of assessors, multiple measurement on variability and generalizability and correlation with clinical patient outcomes.

Unlike aviation, where pilots must undergo certification every year to prove their competency in the aircraft [66], there is no objective assessment and (re)certification of surgeons based on their technical performance in current surgical practice in the Netherlands. In most countries, as in the Netherlands, surgeons apply for periodic recertification by providing proof of a minimum number of surgical procedures in their field and a minimal number of continuing medical education points. This, however, does not necessarily reflect technical proficiency in the execution of said surgical procedures. Since surgery is increasingly prone to new developments and research in which procedures and techniques change over time, the lack of competency assessment is notable. Within the UK, a national training program (LAPCO), in which surgeons were objectively assessed with a PSAT and a GAS tool, has shown to result in improvement of clinical outcomes after laparoscopic colorectal surgery [67]. Multiple surgical training programs utilize some form of competency assessment, but structured (inter)national training programs that embed assessment of surgical skills are still scarce.

To implement training, proctoring and (re)certification, a degree of standardization of surgical procedures is necessary. This is challenging as there are many acceptable surgical variations within any single surgical procedure. In many fields of laparoscopic surgery, there is a lack of evidence and consensus regarding the ‘best surgical technique’. Therefore, it is unknown what steps and elements an objective SQA tool should contain. However, some included studies performed Delphi rounds to agree on the best surgical practice in their field and developed a PSAT based on consensus. This seems to be an appropriate first step towards objective assessment, allowing detailed SQA tools with high level of objectiveness.

Clinical trials investigating new techniques often fail to demonstrate the real benefit of a specific change in a procedure. This may possibly be a result of variation or difference in surgeons proficiency. For example in the field of laparoscopic right hemicolectomy, studies have focused on the comparison of D3 lymphadenectomy versus D2 lymphadenectomy. However, whether a D2 or even D3 implies the same level of lymphadenectomy among or within these respective studies is subject of debate [68]. Also, randomized clinical trials comparing different laparoscopic techniques (ROLARR, ALaCaRT) have not used quality control of surgery which may have influenced the outcomes [69, 70]. The COLOR 3 study (an international randomized clinical trial comparing laparoscopic with transanal total mesorectal excision) is one of the first trials that performs video-based quality control using a CAT to either assess the competence of a potential participating center in a pretrial phase, and to control the quality throughout the study by assessment of videotapes of the surgery of all included patients [59, 71]. Robust competency assessment ensures quality of trials and allows for better comparison of surgical procedures in a research setting.

This systematic review has some limitations. The present study included only tools assessing technical skills. Since it is obvious that teamwork, leadership, decision-making, situational awareness and communication are as important to the whole surgical process as surgical technical skills, these non-technical skills have rightly gained a lot of focus in the last years [72]. The black box in the operating room is an example of an analytical data platform that could be accepted to aid process optimization and, as a result, to also improve the non-technical skills of the operating theatre team [73]. In the future, the combination of assessing both technical and non-technical skills should become important. In addition, a limitation is that we have only focused on video-based SQA tools and not on the live assessment of technical skills. We deliberately chose to do this because we believe that it is the way forward. Thanks to current use of minimally invasive techniques, it is relatively simple to record operations, which has the benefit of enabling postoperative and remote assessment.

The assessments were all based on videotaped cases, which has the advantage of allowing many assessors to evaluate the same procedure at the same time. Furthermore, independent scoring allows assessors to rewind a surgical step for repeated watching while remaining blind to the surgeon's identity and level of expertise, resulting in a more objective evaluation. On the other hand, video-based examination, might be labor intensive, time-consuming and prone to bias. AI could be used in the future to automatically and rapidly identify crucial steps and operational tasks without the assistance of reviewers. Although only one study was included in this review that described the use of AI to assess videos of laparoscopic surgery in the clinical setting [28], a systematic review published in 2022 has already found 66 studies detailing the application of AI for technical skill assessment in surgery [10]. In the near future, probably more developments will be put into practice.

Next to laparoscopic surgery, SQA tools could be of great use in quality control of minimally invasive robotic surgery which is rapidly emerging and will probably play a more important role in the next decade [74]. Since endoscopic and robotic procedure also make use of a camera, these approaches seem suitable for assessment using video-based SQA tools. For the robotic procedures the laparoscopic SQA tools can be used as these approaches are essentially similar and for the endoscopic procedures it would certainly make sense to develop separate SQA tools. However, objective video-based quality assessment of open surgery might be more challenging since adding a camera that provides a good and clear overview of the operation field might bring practical difficulties. In future research, it will be key that there is a focus on the use of SQA tools that incorporate both procedure-specific assessment as well as general skills. Future studies should ideally use tools that are developed using the Delphi technique, implement training for the assessors, use multiple measures of inter-rater reliability, internal consistency and generalizability, validate their tool by clinical outcomes and focus on the interpretation and future use such as cut-off values.

## Conclusion

This systematic review evaluated a total of 41 different video-based SQA tools for technical skills used in 9 fields of laparoscopic surgery. These tools could be divided in global assessment scales, error-based scales, procedure-specific assessment tools and artificial intelligence machine learning. This study shows that well validated SQA tools enable objective assessment of technical skills of a surgeon, with major relevance for patient outcomes. Global assessment scales combined with a procedure-specific assessment tool could have the greatest potential for the use of education, research and certification.

## Supplementary Information

Below is the link to the electronic supplementary material.

Supplementary file1 (DOCX 21 KB)

Supplementary file2 (DOCX 81 KB)
