# Supplementary material for: Video-based tools for surgical quality assessment of technical skills in laparoscopic procedures: a systematic review
Source: Surg Endosc. 2023 Apr 26;37(6):4279–97. doi: 10.1007/s00464-023-10076-z (PMC10234871; doi:10.1007/s00464-023-10076-z)
Supplement: Supplementary file 1 — Supplementary file1 (DOCX 21 KB) [file 464_2023_10076_MOESM1_ESM.docx]

**Supplementary Table 1:** Search strategies for PubMed, Embase.com and Web of Science

**Supplementary Table 1a** Search strategy for PubMed (1 September 2022).

| **Search** | **Query** | **Items found** |
| --- | --- | --- |
| #1 | Search **"surgery" [Subheading] OR "Surgical Procedures, Operative"[Mesh] OR "Surgeons"[Mesh] OR "surger*"[tiab] OR "surgical*"[tiab] OR "surgeon*"[tiab] OR "operation*"[tiab] OR "operative*"[tiab] OR "intraoperative*"[tiab]** | 5248406 |
| #2 | Search **"tool*"[tiab] OR "instrument*"[tiab] OR "scale*"[tiab] OR "score*"[tiab] OR "scoring*"[tiab]** | 2922832 |
| #3 | Search **"Outcome and Process Assessment, Health Care"[Mesh:NoExp] OR "Outcome Assessment, Health Care"[Mesh:NoExp] OR "Process Assessment, Health Care"[Mesh] OR "assess*"[tiab] OR "grading*"[tiab] OR "grade*"[tiab] OR "laparoscopic skill*"[tiab] OR "technical skill*"[tiab] OR "surgical skill*"[tiab] OR "score*"[tiab] OR "scoring*"[tiab]** | 4664426 |
| #4 | Search **"Quality Assurance, Health Care"[Mesh:NoExp] OR "Quality of Health Care"[Mesh:NoExp] OR "Quality Improvement"[Mesh] OR "Quality Indicators, Health Care"[Mesh] OR "Professional Competence"[Mesh] OR "quality instrument*"[tiab] OR "quality assuranc*"[tiab] OR "objective*"[tiab] OR "performan*"[tiab] OR "competenc*"[tiab]** | 4119312 |
| #5 | Search **"Video Recording"[Mesh] OR "Videotape Recording"[Mesh] OR "video*"[tiab] OR "film*"[tiab]** | 376275 |
| #6 | Search **"Laparoscopy"[Mesh:NoExp] OR "laparoscop*"[tiab] OR "endoscop*"[tiab] OR "angioscop*"[tiab] OR "arthroscop*"[tiab] OR "bronchoscop*"[tiab] OR "colposcop*"[tiab] OR "culdoscop*"[tiab] OR "cystoscop*"[tiab] OR "hysteroscop*"[tiab] OR "laryngoscop*"[tiab] OR "mediastinoscop*"[tiab] OR "neuroendoscop*"[tiab] OR "thoracoscop*"[tiab] OR "ureteroscop*"[tiab]** | 508726 |
| #7 | #1 AND #2 AND #3 AND #4 AND #5 AND #6 | 2118 |

**Supplementary Table 1b** Search strategy for Embase.com (1 September 2022).

| **Search** | **Query** | **Items found** |
| --- | --- | --- |
| #1 | 'surgery'/exp OR 'surgeon'/exp OR surger*:ti,ab,kw OR surgical*:ti,ab,kw OR surgeon*:ti,ab,kw OR operation*:ti,ab,kw OR operative*:ti,ab,kw OR incisi*:ti,ab,kw OR extracti*:ti,ab,kw OR excisi*:ti,ab,kw OR invasive*:ti,ab,kw OR restorati*:ti,ab,kw | 7798502 |
| #2 | 'tool*':ti,ab,kw OR 'instrument*':ti,ab,kw OR 'scale*':ti,ab,kw OR 'score*':ti,ab,kw OR 'scoring*':ti,ab,kw | 4032431 |
| #3 | 'outcome assessment'/de OR 'health care quality'/exp OR 'assess*':ti,ab,kw OR 'grading*':ti,ab,kw OR 'grade*':ti,ab,kw OR 'laparoscopic skill*':ti,ab,kw OR 'technical skill*':ti,ab,kw OR 'surgical skill*':ti,ab,kw OR 'score*':ti,ab,kw OR 'scoring*':ti,ab,kw | 9315730 |
| #4 | 'health care quality'/exp OR 'quality control'/exp OR 'professional competence'/exp OR 'quality instrument*':ti,ab,kw OR 'quality assuranc*':ti,ab,kw OR 'objective*':ti,ab,kw OR 'performan*':ti,ab,kw OR 'competenc*':ti,ab,kw | 8741930 |
| #5 | 'videorecording'/exp OR 'endoscopic video camera'/exp OR 'videotape'/exp OR 'video*':ti,ab,kw OR 'film*':ti,ab,kw | 428862 |
| #6 | 'laparoscopy'/exp OR 'laparoscopic surgery'/exp OR 'laparoscop*':ti,ab,kw OR 'endoscop*':ti,ab,kw OR 'angioscop*':ti,ab,kw OR 'arthroscop*':ti,ab,kw OR 'bronchoscop*':ti,ab,kw OR 'colposcop*':ti,ab,kw OR 'culdoscop*':ti,ab,kw OR 'cystoscop*':ti,ab,kw OR 'hysteroscop*':ti,ab,kw OR 'laryngoscop*':ti,ab,kw OR 'mediastinoscop*':ti,ab,kw OR 'neuroendoscop*':ti,ab,kw OR 'thoracoscop*':ti,ab,kw OR 'ureteroscop*':ti,ab,kw | 816046 |
| #7 | #1 AND #2 AND #3 AND #4 AND #5 AND #6 | 4353 |
| #8 | #7 NOT ('conference abstract'/it OR 'conference paper'/it OR 'conference review'/it) | 2755 |

**Supplementary Table 1c** Search strategy for Web of Science (1 September 2022).

| **Search** | **Query** | **Items found** |
| --- | --- | --- |
| #1 | **TOPIC:** (“surger*” OR “surgical*” OR “surgeon*” OR “operation*” OR “operative*” OR “incisi*” OR “extracti*” OR “excisi*” OR “invasive*” OR “restorati*”) | 4704370 |
| #2 | **TOPIC:** (“tool*” OR “instrument*” OR “scale*” OR “score*” OR “scoring*”) | 5472945 |
| #3 | **TOPIC:** (“assess*” OR “grading*” OR “grade*” OR “laparoscopic skill*” OR “technical skill*” OR “surgical skill*” OR “score*” OR “scoring*”) | 6109218 |
| #4 | **TOPIC:** (“quality instrument*” OR “quality assuranc*” OR “objective*” OR “performan*” OR “competenc*”) | 7287039 |
| #5 | **TOPIC:** (“video*” OR “film*”) | 1703758 |
| #6 | **TOPIC:** (“laparoscop*” OR “endoscop*” OR “angioscop*” OR “arthroscop*” OR “bronchoscop*” OR “colposcop*” OR “culdoscop*” OR “cystoscop*” OR “hysteroscop*” OR “laryngoscop*” OR “mediastinoscop*” OR “neuroendoscop*” OR “thoracoscop*” OR “ureteroscop*”) | 540669 |
| #7 | #1 AND #2 AND #3 AND #4 AND #5 AND #6 | 1619 |
