# Supplementary material for: Video-based tools for surgical quality assessment of technical skills in laparoscopic procedures: a systematic review
Source: Surg Endosc. 2023 Apr 26;37(6):4279–97. doi: 10.1007/s00464-023-10076-z (PMC10234871; doi:10.1007/s00464-023-10076-z)
Supplement: Supplementary file 2 — Supplementary file2 (DOCX 81 KB) [file 464_2023_10076_MOESM2_ESM.docx]

**Supplementary Table 2:** Evidence of validity of the included studies per domain based on the validity evidence score list from Table 1.

| Kind of assessment | Article | Content | Response process | Internal structure | Relations to other variables | Consequences | Total |
| --- | --- | --- | --- | --- | --- | --- | --- |
| Global assessment scale (GAS) | Varban [61] | 2 | 1 | 0 | 3 | 0 | **6** |
|  | Varban 62] | 2 | 1 | 0 | 3 | 1 | **7** |
|  | Chhabra [21] | 2 | 0 | 0 | 3 | 2 | **7** |
|  | Fecso [26] | 3 | 1 | 0 | 3 | 2 | **9** |
|  | Goderstad [29] | 3 | 0 | 1 | 2 | 0 | **6** |
|  | Scally [55] | 2 | 1 | 1 | 3 | 1 | **8** |
|  | Kramp [42] | 2 | 2 | 3 | 2 | 3 | **12** |
|  | Koehler [40] | 3 | 2 | 2 | 0 | 1 | **8** |
|  | Kramp [41] | 2 | 2 | 2 | 1 | 1 | **8** |
|  | Kasparian [36] | 1 | 1 | 1 | 2 | 1 | **6** |
|  | Matsuda [47] | 3 | 1 | 1 | 0 | 0 | **5** |
|  | Birkmeyer [3] | 3 | 1 | 0 | 3 | 2 | **9** |
|  | Koehler [39] | 3 | 2 | 2 | 1 | 2 | **10** |
|  | Oestergaard [50] | 2 | 1 | 1 | 1 | 0 | **5** |
|  | Herati [33] | 2 | 1 | 1 | 1 | 0 | **5** |
|  | Larsen [44] | 3 | 1 | 1 | 1 | 2 | **8** |
|  | Aggarwal [15] | 2 | 1 | 3 | 2 | 1 | **9** |
|  | Aggarwal [16] | 2 | 0 | 2 | 3 | 2 | **9** |
|  | Chang [19] | 2 | 0 | 1 | 1 | 0 | **4** |
|  | Vassiliou [63] | 2 | 2 | 2 | 1 | 2 | **9** |
|  | Shime [56] | 3 | 2 | 3 | 1 | 2 | **11** |
| Error-based assessment scale (EBAS) | Fecso [26] | 3 | 1 | 0 | 3 | 2 | **9** |
|  | Foster [27] | 2 | 0 | 1 | 3 | 1 | **7** |
|  | Husslein [34] | 2 | 2 | 2 | 1 | 2 | **9** |
|  | Bonrath [8] | 2 | 1 | 2 | 3 | 1 | **9** |
|  | Miskovic [49] | 2 | 2 | 2 | 2 | 1 | **9** |
|  | Tang [58] | 2 | 2 | 0 | 3 | 0 | **7** |
| Procedure-specific assessment tool (PSAT) | Haug [32] | 3 | 0 | 3 | 0 | 2 | **8** |
|  | Sirimanna [57] | 2 | 2 | 2 | 2 | 2 | **10** |
|  | Chevallay [20] | 1 | 2 | 2 | 0 | 2 | **7** |
|  | Kurashima [43] | 3 | 2 | 1 | 3 | 2 | **11** |
|  | Harris [31] | 3 | 2 | 1 | 0 | 1 | **7** |
|  | Kobayashi [38] | 2 | 1 | 0 | 3 | 0 | **6** |
|  | Dixon [24] | 2 | 1 | 0 | 0 | 0 | **3** |
|  | Crochet [22] | 3 | 1 | 1 | 1 | 2 | **8** |
|  | Han [30] | 3 | 1 | 2 | 3 | 1 | **9** |
|  | Stulberg [1] | 2 | 3 | 2 | 3 | 2 | **12** |
|  | Varban 60] | 1 | 1 | 0 | 3 | 1 | **6** |
|  | Curtis [2] | 3 | 1 | 2 | 3 | 3 | **12** |
|  | Tsai [59] | 3 | 0 | 3 | 0 | 0 | **6** |
|  | Ki Bum Park [51] | 2 | 1 | 0 | 2 | 0 | **5** |
|  | Savran [54] | 3 | 1 | 2 | 1 | 3 | **10** |
|  | Jensen [35] | 3 | 0 | 0 | 0 | 0 | **3** |
|  | Petersen [52] | 3 | 2 | 2 | 1 | 3 | **11** |
|  | Champagne [18] | 3 | 2 | 2 | 2 | 3 | **12** |
|  | Deal [23] | 2 | 2 | 2 | 1 | 1 | **8** |
|  | Goderstad [29] | 3 | 0 | 1 | 2 | 0 | **6** |
|  | Kramp [42] | 2 | 2 | 3 | 2 | 3 | **12** |
|  | Poudel [53] | 3 | 2 | 1 | 2 | 2 | **10** |
|  | Mackenzie [46] | 2 | 2 | 0 | 3 | 1 | **8** |
|  | Miskovic [48] | 3 | 1 | 3 | 2 | 3 | **12** |
|  | Zevin [64] | 3 | 2 | 3 | 2 | 2 | **12** |
|  | Oestergaard [50] | 2 | 1 | 1 | 1 | 0 | **5** |
|  | Palter [9] | 3 | 2 | 1 | 1 | 2 | **9** |
|  | Herati [33] | 2 | 1 | 1 | 1 | 0 | **5** |
|  | Larsen [44] | 3 | 1 | 1 | 1 | 2 | **8** |
|  | Eubanks [25] | 3 | 1 | 1 | 2 | 2 | **9** |
|  | Beckmann [17] | 2 | 1 | 0 | 0 | 2 | **5** |
| Artificial Intelligence (AI) | Kitaguchi [37] | 3 | 3 | 0 | 1 | 1 | **8** |
|  | Lavanchy [45] | 3 | 3 | 0 | 1 | 0 | **7** |
|  | Ganni [28] | 1 | 2 | 1 | 2 | 2 | **8** |
